# Overcoming roadblocks for *in vitro* nurseries in plants: induction of meiosis

**DOI:** 10.3389/fpls.2023.1204813

**Published:** 2023-06-02

**Authors:** Tanner M. Cook, Daniel Isenegger, Somak Dutta, Sareena Sahab, Pippa Kay, Siddique I. Aboobucker, Eva Biswas, Seth Heerschap, Basil J. Nikolau, Liang Dong, Thomas Lübberstedt

**Affiliations:** ^1^ Iowa State University, Department of Agronomy, Ames, IA, United States; ^2^ Agriculture Victoria, Agribio, La Trobe University, Melbourne, VIC, Australia; ^3^ Iowa State University, Department of Statistics, Ames, IA, United States; ^4^ Iowa State University, Department of Electrical and Computer Engineering, Ames, IA, United States; ^5^ Iowa State University, Roy J. Carver Department of Biochemistry, Biophysics, and Molecular Biology, Ames, IA, United States

**Keywords:** meiosis induction, *in vitro* biology, *in vitro* nurseries, high-throughput detection, plant breeding and biotechnology

## Abstract

Efforts to increase genetic gains in breeding programs of flowering plants depend on making genetic crosses. Time to flowering, which can take months to decades depending on the species, can be a limiting factor in such breeding programs. It has been proposed that the rate of genetic gain can be increased by reducing the time between generations by circumventing flowering through the *in vitro* induction of meiosis. In this review, we assess technologies and approaches that may offer a path towards meiosis induction, the largest current bottleneck for *in vitro* plant breeding. Studies in non-plant, eukaryotic organisms indicate that the *in vitro* switch from mitotic cell division to meiosis is inefficient and occurs at very low rates. Yet, this has been achieved with mammalian cells by the manipulation of a limited number of genes. Therefore, to experimentally identify factors that switch mitosis to meiosis in plants, it is necessary to develop a high-throughput system to evaluate a large number of candidate genes and treatments, each using large numbers of cells, few of which may gain the ability to induce meiosis.

## 1 Introduction to *in vitro* nurseries

**Figure 1:**
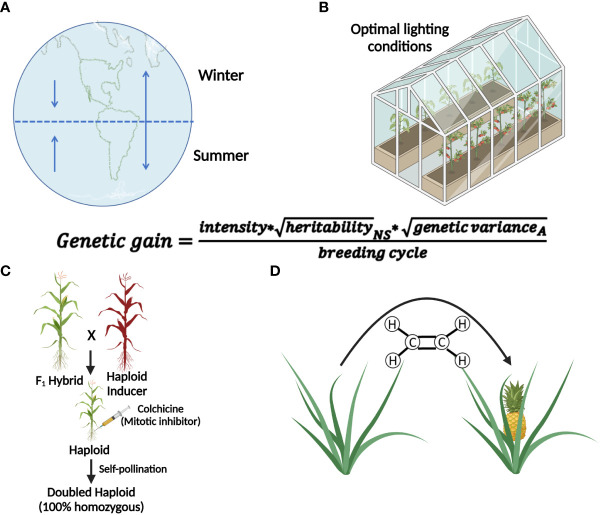
Methods that accelerate breeding cycles or generation time of plants. The genetic gain equation provides the basis for understanding how the use of each technique leads to genetic gain **(A)** The use of winter nurseries to capitalize on proximity to the equator or opposite hemispheric seasons, increase diurnal photoperiods, and warmer temperatures to increase the number of growing seasons per year. **(B)** Speed-breeding techniques that utilize optimal lighting conditions to induce early flowering to decrease breeding cycle times. **(C)** Doubled-haploid technology reduces the time needed to develop a homozygous line. **(D)** Use of chemicals (e.g. ethylene) to induce early flowering and fruit development, decreasing breeding cycle time. Created with BioRender.com.

Globally the number of undernourished people is expected to increase to 840 million by 2030 (FAO et al, 2020). Even though we need to produce more food in the future, the current levels of food production are at risk as climate change has the potential to disrupt food availability (Brummer et al., 2011; De La Fuente et al., 2013; Brown et al., 2015; US Embassy and Consulate in Italy, 2022). Innovative breeding techniques to improve food security to increase genetic gains are needed. Genetic gain is associated with selection intensity, heritability, genetic variance, and the time needed for a breeding cycle (**Figure 1**; Li et al., 2018). Several breeding methods and technologies have been developed to increase genetic gain by reducing the time needed to complete a breeding cycle, and these include winter nurseries, doubled-haploids (Geiger, 2009; De La Fuente et al., 2013; Boerman et al., 2020), speed breeding (Watson et al., 2018; Jähne et al., 2020), gene editing or gene expression regulation (Gao et al., 2020; Pan et al., 2022; reviewed in Zhang et al., 2018), marker-assisted selection (Karunarathna et al., 2021; López-Malvar et al., 2021; Tibbs Cortes et al., 2021), genomic selection (Bhat et al., 2016; López-Malvar et al., 2021), phytohormonal induction of early flowering (Espinosa et al., 2017), and combination of doubled-haploids with other breeding strategies such as gene editing during haploid induction and genomic prediction (Kelliher et al., 2019; Wang et al., 2020). These technologies focus on reducing the number of generations needed to develop a line, or reducing the time to flowering and seed production, thereby allowing more generations per unit of time. Technologies such as speed breeding and hormone manipulation provide earlier flowering times but are only available for a limited number of crop species, (Iqbal et al., 2017; Watson et al., 2018; Jähne et al., 2020), and they still require that plants produce floral organs and gametes for sexual reproduction.

As a paradigm shift, Murray et al. (2013) and De La Fuente et al. (2013) suggested the concept of a cell-based *in vitro* breeding system (called *in vitro* nurseries; IVNs). In IVNs, breeding cycle time could be substantially reduced by enabling rapid cell-level breeding cycles, without the need for flowering. Somatic tissue from parental lines could be cultured and challenged to induce haploid cells after recombination without gametophyte development (will be referred to as artificial gametes throughout the text), these cells can then be fused artificially to develop sexual pairing *in vitro*. In addition to time, IVNs will significantly reduce required field space and avoid exposure to environmental risks in field settings. The benefits of IVNs are of particular interest for species with a long generation time. Some woody species do not produce flowers for more than 30 years (Hackett, 1985). For example, poplar trees provide many ecosystem services such as phytoremediation, substrate for biofuels, and other bioproducts (Zalesny et al., 2020), but can take 10 years to flower (Hsu et al., 2006). Coffee trees do not flower until the second year, and it is not until the third year that they reach maturity (Santos et al., 2019). Even in annual crops such as maize, where two generations per year are routinely completed using winter nurseries, more generations per year would increase the annual genetic gain significantly.

**Figure 2:**
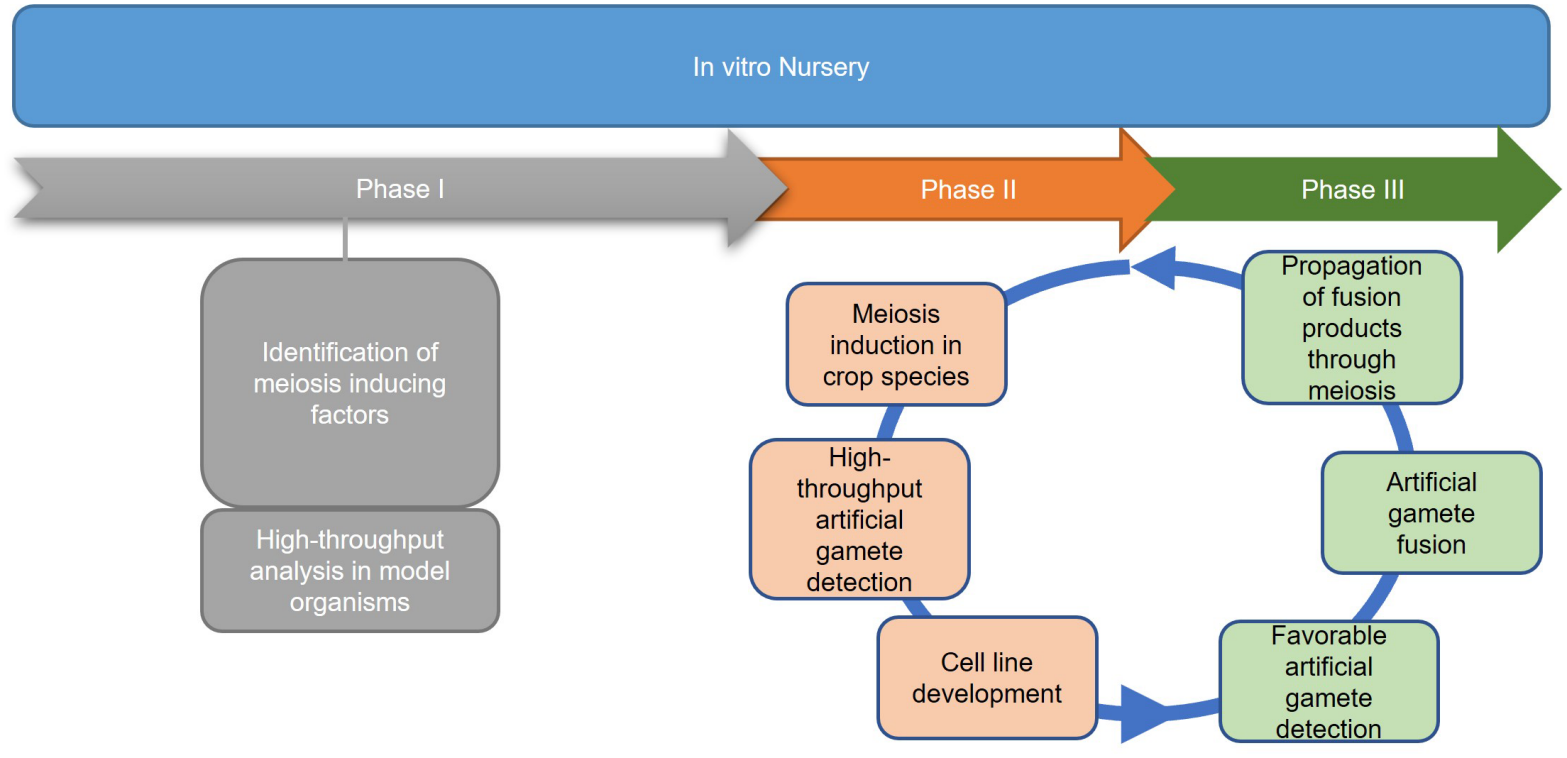
Three proposed phases of an *in vitro* nursery. Phase I emphasizes the identification of meiosis-inducing factors as a major obstacle in establishing IVNs. Identification of the meiosis-inducing factors needs a quantitative, high throughput assay to detect meiosis. Phase II involves the development of the nursery in the context of a specific crop species, using the meiosis-inducing factors identified in Phase I and developing haploid cell lines for the selection of desirable traits. Phase III identifies haploid cell lines that are expressing desirable traits by the use of markers and traditional breeding tools. After diploidization, the products are propagated by mitosis for further cycling through Phases II and III.

Successful implementation of IVNs will require systematically overcoming a variety of bottlenecks. We anticipate three distinct phases (**Figure 2**). Phase I addresses the main bottleneck: meiosis induction (or meiosis-like recombination followed by reductional division) from somatic vegetative tissue, which currently is unavailable in plants. We assume that there will be a need to evaluate a potentially large number of genes, external treatments, and their combinations before identifying a path for meiosis induction in plants. Thus, an assay for high-throughput and low-cost screening of candidate genes or treatments for meiosis induction is needed. The other two phases are based on the successful development of protocols for meiosis induction. Phase II addresses artificial gamete formation and identification. Identifying and isolating these artificial gametes in a mixture consisting primarily of somatic cells will be critical for manipulation in the next phase. Phase III includes the assessment of induced artificial gametes that carry favorable alleles using genomic selection methods. This can only be done after artificial gametes have been isolated and developed into cell lines. Only then, can a sample of cells from each line be sacrificed for DNA isolation and genotyping for genomic selection. Further, this phase includes the fusion of selected artificial gametes to generate diploid cells, as a starting point for the next generation in IVNs. In this review, we will assess the concept of a cell-based *in vitro* breeding system, which circumvents the need for flowering (De la Fuente et al., 2013; Murray et al., 2013). The overall objective of this paper is to investigate the feasibility of Phase I through the (i) identification of bottlenecks and uncertainties (ii) while proposing possible solutions, and thus (iii) providing a starting point for the development of IVN technologies.

## 2 Eukaryotic meiosis

### 2.1 Meiosis in plants

Sex is a fundamental process shared among eukaryotes (Colnaghi et al., 2020), with meiosis being a key step to generating variation by recombining genomes. Meiosis consists of DNA replication followed by two divisions that reduce the genome size by half (Mercier et al., 2015). During meiosis, chromosomes recombine via crossovers (COs), a mechanism to reshuffle genes and respective physically linked alleles on a chromosome (Mercier et al., 2015). The major obstacle in establishing IVNs in plants is the inability to induce meiosis outside of the male or female reproductive cell structures of the flower. To be practical for IVNs, *in vitro* meiosis induction has to be based on a limited number of factors to enable a practical, routine application for artificial gamete formation.

**Figure 3:**
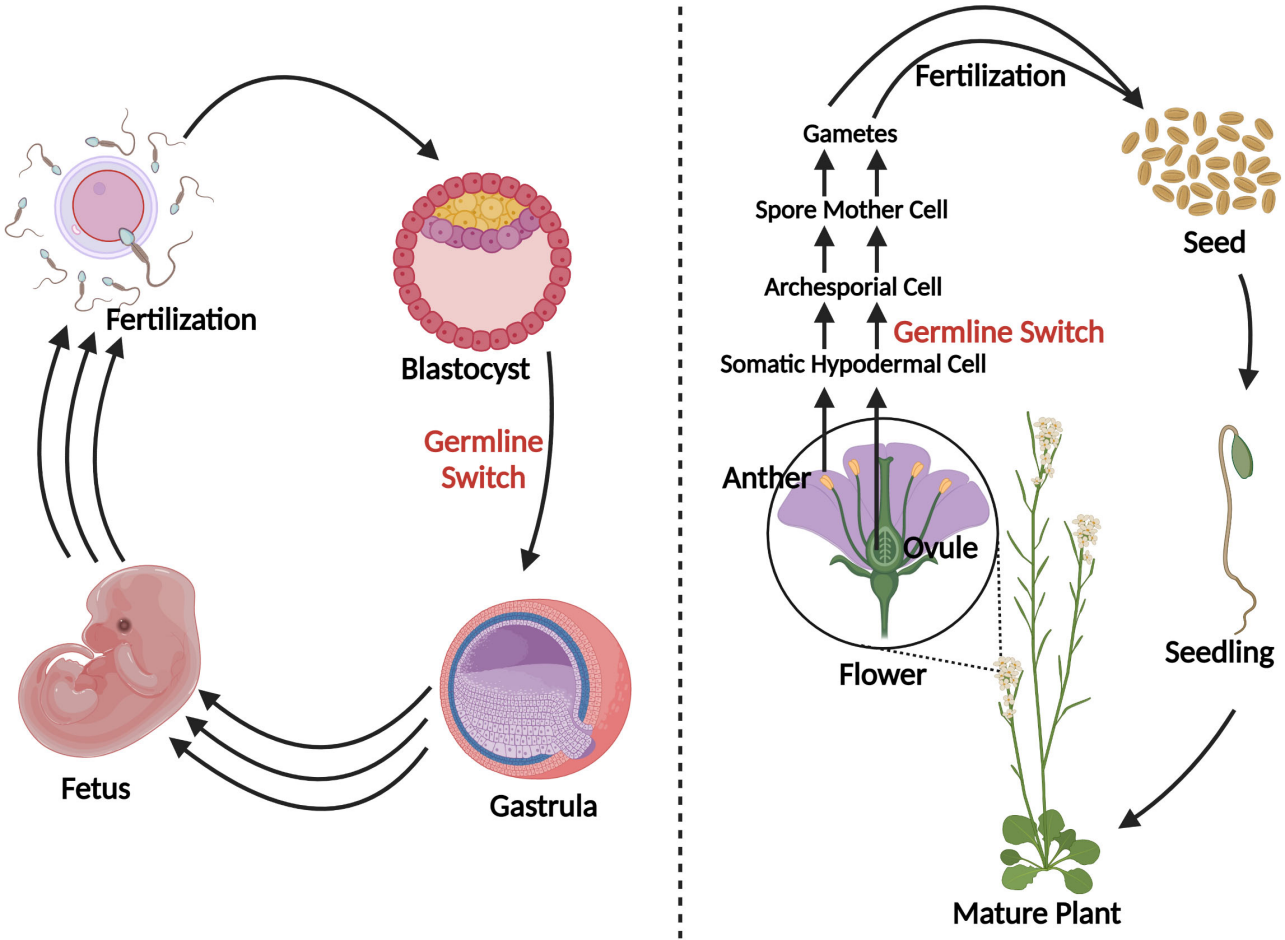
A schematic comparison of germline development in humans vs. plants. Blastocyst formation takes approximately five days (Popovic et al., 2021), after which germline in humans develops and can be detected as early as the beginning of gastrulation (Wen and Tang, 2019). Three sets of arrows indicate additional developmental processes that were not discussed. In plants, germline development is much later in a plant’s life cycle, occurring during flowering. Somatic hypodermal cells divide to develop an archesporial cell, which is considered the origin of the germline (Zhou et al., 2017). Archesporial cells will then form spore mother cells which undergo meiosis to develop gametes. Created with BioRender.com.

In contrast to the predetermined germline of animals, the transition from vegetative to reproductive growth in plants occurs later in development where archesporial cells are generated from primordia, beginning the plant germline (Zhou et al., 2017). Differences between plant and human germline development have been outlined in **Figure 3**. In angiosperms, gametogenesis is a highly conserved process and occurs within specialized tissues of the anther and the ovule. The production of gametes proceeds in two steps: sporogenesis, followed by gametogenesis. In the anther, hypodermal archesporial cells divide to produce outer primary parietal cells which become somatic tissues, and inner primary sporogenous cells which will then divide mitotically to become microspore mother cells that undergo meiosis (Lora and Hormaza, 2021). Ovule initiation arises from the medial meristem tissue within the carpel. Immediately following ovule initiation three distinct regions arise: funiculus, chalaza, and nucellus. The nucellus gives rise to the megaspore mother cell which undergoes meiosis to generate four megaspores, three of which degrade leaving a single functional megaspore (Lora and Hormaza, 2021).

### 2.2 Controlling meiosis in other eukaryotes

Since the underlying evolutionary path of sexual reproduction is thought to have evolved only once in eukaryotes (Goodenough and Heitman, 2014), there is insight to gain from other non-plant, eukaryotic species. For example, Medrano et al. (2016) found that human somatic cells can be converted into germline-like cells with the ectopic expression of six genes. These genes, *PRDM1*, *PRDM14*, *LIN28A*, *DAZL*, *VASA*, and *SYCP3*, have conserved regions in many plant species and the products have been shown to have important functions in such processes such as the repression of transposable elements, nucleic acid binding, and stem cell maintenance in human meiotic processes (Bateman, 2019; Howe et al., 2020). About 1% of these germline-like cells were able to complete meiosis (Medrano et al., 2016). Further, overexpression of human CD61 (integrin-β3) in canine adipose-derived mesenchymal stem cells led to the upregulation of markers for primordial germ-like cells (Fang et al., 2017). Vernet et al. (2020) suggested that the exogenous application of retinoic acid may force meiosis induction in mice. *In vitro* mouse studies of spermatogonia overexpressing telomerase catalytic component, mTERT, resulted in cells that could be induced to undergo meiosis *in vitro*, with the application of stem cell factor (Feng et al., 2002; Griswold, 2005; Riou et al., 2005). This outcome suggests that sex cells can be developed without structure-specific nurse cells, which is encouraging in the case of IVNs. In *S. cerevisiae*, antisense transcription was found to control meiotic cell entry by regulating IME4 (Initiator of Meiosis 4), an RNA methyltransferase (Hongay et al., 2006). This gene is also expressed in the testes and ovaries of *Drosophila* (Hongay and Orr-Weaver, 2011). In *Arabidopsis thaliana*, MTA (mRNA adenosine methylase), which is a homolog of IME4, was found to be essential for embryogenesis (Zhong et al., 2008). In addition to IME4, nutritional stress can also induce meiosis in yeast (Mata et al., 2002). Taken together, such studies in other eukaryotic species suggest that meiosis or meiotic precursors can be artificially induced and that this may also be achievable with plants.

### 2.3 Plant genetic factors involved in meiosis induction

In plants, a limited number of genetic factors have been identified to play a role in meiosis induction or early meiotic processes by studying aberrant phenotypes presented by mutant alleles. **Table 1** summarizes the genes that have been found in previous studies. Maize AMEIOTIC 1 (AM1) is required for meiotic progression while it is also likely required for meiosis initiation as premeiotic cells with *am1* mutations led to mitosis instead of meiosis (Pawlowski et al., 2009). The SWI(SWITCH1)/DYAD protein is a putative homolog of AM1 in *Arabidopsis*, but its role appears to be more important in early meiosis instead of initiation. Consistent with this, SWI/DYAD maintains chromatid cohesion during meiosis as a WINGS APART-LIKE antagonist (Pawlowski et al., 2009; Yang et al., 2019). Evidence for the role of FEHLSTART (FST), a basic helix-loop-helix protein, in meiosis is shown with early meiotic entry in *Arabidopsis* mutants, and these mutants also show meiotic asynchrony (Li et al., 2015). KRP4, KRP6, and KRP 7 (KIP-RELATED PROTEIN 4,5,6) as well as RETINOBLASTOMA RELATED1 (RBR1), prevent the formation of supernumerary meiocytes from forming next to an already existing meiocyte while the repression of WUSCHEL (WUS) by RBR1 allows entry into meiosis (Zhao et al., 2017). An RNA-helicase (RH17) was found to play a role in reproduction as supernumerary reproductive cell lineages developed at a rate of over 20% in lines that were heterozygous for an *rh17* mutant allele (Stein et al., 2021). Evidence for potential clues in phase change induction is further supported by the *Mitosis instead of Meiosis* phenotype in rice and *Arabidopsis*, where mutations in only three genes prevent meiotic cell entry and instead meiocytes in the gametophyte undergo mitosis (Mieulet et al., 2016). This phenotype can be developed with triple mutations in *REC8* and *OSD1* in combinations with either *SPO11* or *PRD1,2,3* mutants in *Arabidopsis* and with the combination of *REC8*, *PAIR1*, and *OSD1* mutations in rice (Mieulet et al., 2016). These previous studies lay a strong foundation on which we can build an understanding of meiosis induction in plants.

**Table 1:** Genes involved in meiotic entry and regulation.

Gene/Nucleic Acid	Meiotic Role*	Species	Source
*SWI1/DYAD/Am1*	Cohesion, progression, and initiation	Arabidopsis/Rice/Maize	Pawlowski et al., 2009; Yang et al., 2019
*MEL2*	Regulates premeiotic G1/S-phase transition and synchrony	Rice	Nonomura et al., 2011
*SPL/NZZ*	Meiotic entry, meiotic fate acquisition, and ovule development	Rice/Arabidopsis	Wei et al., 2015; Ren et al., 2018
*RBR1*	WUS repression leading to meiotic entry	Arabidopsis	Zhao et al., 2017
*FST*	Meiotic entry and synchrony	Arabidopsis	Li et al., 2015
*MIL1*	Initiation and differentiation	Rice	Hong et al., 2012
*AGO9/AGO104*	Cell fate specification	Arabidopsis/Maize	Olmedo-Monfil et al., 2010; Singh et al., 2011
*DTM1*	Tapetum development and meiotic prophase 1 progression	Rice	Yi et al., 2012
*MEI1*	Meiotic-specific DNA repair	Arabidopsis	Mathilde et al., 2003
*CDC45*	Correct meiotic division progression	Arabidopsis	Stevens et al., 2004
*XRI1*	Meiotic DNA repair	Arabidopsis	Dean et al., 2009

*All homologous genes may not have all of the roles listed.

Many of these genes are reviewed in more detail by Mercier et al. (2015) and Wang et al. (2021).

### 2.4 Plant hormonal and environmental factors

Hormonal cues from surrounding somatic tissue in the developing gametophyte also affect meiotic processes. Auxin signaling is likely to provide cues for the differentiation of egg cells vs. synergid cells in the egg apparatus (Sun et al., 2021). Auxin and brassinosteroids are important factors in meiocyte development as peak expression in biosynthesis and signaling is found in meiotic anthers (Dhaka et al., 2020). In addition, an auxin gradient appears to play a role in male germ cell development (Zheng et al., 2021). Cytokinin is shown to play a role in meiotic processes as well. For example, cytokinin histidine kinase receptors, AHK2, AHK3, and CRE1, are attributed with the ability to sense environmental cytokinin to create a kinase cascade, while triple knockouts of these three genes, result in cytokinin unresponsive plants (Inoue et al., 2001; Higuchi et al., 2004; Cheng et al, 2013). Loss of function with these cytokinin receptors results in female gametophytic lethality but can be recovered via TDNA complement insertions (Higuchi et al, 2004; Cheng et al, 2013). Environmental conditions such as hypoxia and oxidation-reduction have also been shown to induce meiotic fate (Kelliher and Walbot, 2012). Mutations in genes associated with redox reactions, like *MSCA1* (maize), *MIL1* (rice), *ROXY1*, and *ROXY2* (*Arabidopsis*), led to fertility disruptions (Xing and Zachgo, 2008; Hong et al., 2012; Kelliher & Walbot, 2012). Further, a switch from apomeiosis to meiosis occurs with increased oxidative stress treatment in *Boechera* premeiotic ovules (Mateo de Arias et al., 2020). In *Arabidopsis*, retinal was determined to be an endogenous metabolite that plays a role in root organogenesis and root clock functions (Dickinson et al., 2021). Interestingly, TEMPERATURE INDUCED LIPOCALIN (TIL) acts as a retinal binder in plants with protective functions in heat stress, light stress, and oxidative stress (Chi et al., 2009; Boca et al., 2014; Dickinson et al., 2021). Evidence for a stress-mediated switch between meiosis and apomeiosis has been demonstrated (Mateo de Arias et al., 2020), and since retinoic acid may force meiosis induction in mice (Vernet et al., 2020), the closely related retinal may function in stress response in plants, and possibly be of interest to explore for meiotic induction. Moreover, the number of candidate genes and factors for meiosis induction has grown substantially in the past years. However, an efficient test system is needed to determine the relevance of candidate factors in meiosis initiation.

## 3 Tools for meiotic factor testing

### 3.1 Cell-based system

Detailed analyses of specific genetic factors and growth hormones provide a great starting place to begin testing factors as meiotic induction candidates but low induction rates in mammals (Medrano et al., 2016) suggest that a high-throughput system is required to evaluate these candidates.

High-throughput, single-cell culture systems, such as protoplasts, may provide a robust approach to detecting the rare meiotic events induced by multiple factors. Protoplasts are spherical-shaped cells that are devoid of the cell wall, removed by enzymatic digestion, and can provide totipotent homogeneous populations of cells useful for plant genetic improvement studies in some species (Davey et al., 2005; Eeckhaut et al, 2013; Sahab et al., 2019). An important factor for the viability of protoplasts is maintaining osmotic stabilization to prevent cell lysis after cell wall removal (Marx, 2016; Reed and Bargmann, 2021). Protoplast-based platforms can allow for the hybridization of different species via protoplast fusion and plant regeneration (Melchers et al., 1978). Moreover, protoplasts can enable the exploration of signal transduction and metabolic pathways (Sheen, 2001), cell type-specific functions (Petersson et al., 2015; Denyer et al., 2019), and determine the subcellular localization, transport, and interactions of intracellular proteins (Goodman et al., 2004; Zhang et al., 2011). Cellular division and subsequent regeneration from protoplasts have been reported in numerous species with varying levels of efficiency (Nagata and Takebe, 1971; Xu et al., 1982; Shillito et al., 1989; Kiełkowska and Adamus, 2012; Chupeau et al., 2013; Jeong et al., 2021). However, recalcitrance to protoplast regeneration has also been observed across many species and is particularly challenging in monocotyledonous species (Hahne et al., 1989; Xu et al., 2022). There is a range of factors that can influence establishing reliable protoplast transient assays and regeneration protocols (reviewed in Reed and Bargmann, 2021).

### 3.2 *In vitro* meiosis induction testing system using single cells

Using protoplasts allows many cells to be analyzed at one time while also providing the potential to be collected and used in downstream IVN experiments in addition to simple ploidy analysis. With these single cells, two options have been considered for high-throughput screening of meiosis induction. First, protoplasts can be isolated and then challenged to undergo cellular division. Division would then be followed by a meiotic induction treatment from which dividing cells can be reisolated for ploidy-state analysis. This option can be laborious but provides a means for the analysis of introduced genetic factors (Yoo et al., 2007). The second option can utilize dividing callus, which can be treated with meiotic induction factors followed by protoplast isolation for ploidy-state analysis. This system is potentially less laborious and enables efficient testing of exogenous factors, but the assessment of genetic elements would rely on an efficient transformation system. Both options, however, require callus formation as a result of cellular division of which cell lines could be maintained for analysis and subsequent selection. These protoplast-based approaches would also benefit from culture suspension as multiple factors could be tested while easily moving aliquots of cells for processing and systematic treatment application.

Efficient delivery of genetic elements and the induction and detection of meiosis may be difficult to establish in protoplasts, as cell survival, fitness, and division can be impacted by the product of transgenes and mutagenesis. To test genetic factors, DNA delivery into the cells is required. Conventionally, transgenic plants can be generated via the delivery of DNA-encoding gene constructs via microprojectile bombardment or *Agrobacterium-*mediated transformation (Cunningham et al, 2018). Other types of transformation methods can utilize nanoparticles (Cunningham et al, 2018; Mao et al., 2019), electroporation, microinjection, PEG-mediated direct delivery in protoplasts (Yoo et al, 2007), and viral-vectors (Catoni et al., 2018; reviewed by Abrahamian et al., 2020). Alternatively, DNA-free CRISPR/Cas genome editing systems can deploy ribonucleoproteins to cells using similar DNA delivery approaches for targeted mutagenesis of genes or regulatory regions to modulate the expression of genes (Woo et al, 2015; Liu et al., 2020; Ma et al., 2020; Zhang et al., 2021). In protoplasts, transcriptional regulation was also an effective means to control gene expression and could be multiplexed (Pan et al., 2021). Given the potential difficulties of genetic element testing in protoplasts, however, protoplasts derived from callus may be highly suited for evaluating chemical factors which can be simply applied in culture media. For instance, chemical factors have been applied to callus cultures to test cell cycle regulation and ploidy increase (Wan et al., 1989; Elmaghrabi et al., 2017), while hormones and stress factors added to callus culture media have been evaluated to increase metabolite production (Beygi et al., 2021). Interestingly, chemical agents can be assessed and deployed to reduce chromosome number, somewhat like haploids or meiosis-like reductions. For example, decades ago a chloramphenicol antibiotic treatment was shown to reduce chromosomes to a haploid state in root cells of barley seedlings (Yoshida and Yamaguchi, 1973). Caffeine treatments have been shown to induce somatic meiosis-like reductions in *Vicia* root tips (Chen et al., 2000). Meiosis-like reductions have also been observed in somatic embryogenic callus cultures of *Arabidopsis* (Yihua et al., 2001) and non-embryogenic carrot cell culture lines that were considered to be permanently expressed in a meiotic or sporogenous tissue state (Ronchi et al., 1992). These studies indicate the potential for screening and deploying chemical agents on explant tissue sources and in *in vitro* culture for meiosis induction in an IVN system. However, the reliable and efficient development of such approaches likely requires extensive work and validation that may vary across different species (Yan et al., 2017). 

### 3.3 High-throughput fluorescence analysis

**Figure 4:**
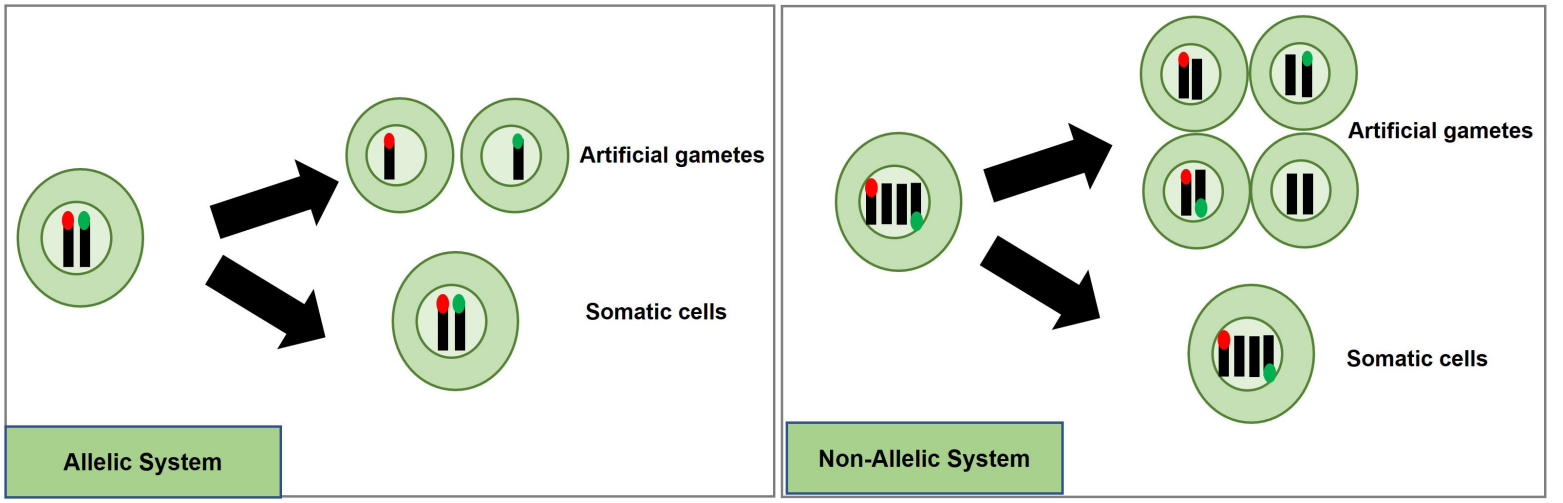
Proposed high-throughput meiosis induction detection tools using bi-fluorescent single cells to track chromosomal segregation. The left panel depicts an allelic system where chromosomal segregation can be identified 1:1. The right panel depicts a non-allelic system where chromosomal segregation can be detected with 50% less efficiency. Red and green dots represent different fluorescent markers on a chromosome, arrows indicate a treatment, and cells right of the arrows are the potential products after treatment, either artificial gametes (if meiosis was induced) or somatic cells (if meiosis was not induced).

A potentially efficient method to detect and quantify meiosis induction with callus-derived protoplasts is through the use of a transgenic, bi-fluorescent system to track chromosomal segregation after meiotic cell division. By utilizing a dual marker system, an assay to detect the induction of meiosis can be achieved based on the presence or absence of fluorescence signals in cells through fluorescence-activated cell sorting (FACS) instruments (Bargmann and Birnbaum, 2010; Borges et al., 2012; Ortiz-Ramírez et al., 2018). DNA content analysis using FACS or flow cytometry can also be used to determine artificial gametes (haploid) and somatic cells (diploid), but traditional stains such as 4’,6-diamidino-2-phenylindole and propidium iodide inefficiently pass through intact cell membranes (Wallberg et al., 2016), while there are other commercially available stains for DNA analysis of live cells, these will have to be controlled for and considered when testing meiotic candidates. These factors present difficulties for further downstream uses in IVN’s and are the basis for the fluorescent system development suggestion. In the proposed fluorescent system, two different fluorescent single-copy reporter genes such as RFP or GFP can be integrated into the genome either in allelic or non-allelic positions (**Figure 4**) and detected without the need for DNA stains.

Generating a genotype with two different markers in allelic positions is more complex than for non-allelic markers. For example, one way to obtain different marker genes in allelic positions would be to establish a homozygous, fluorescent marker line, and use gene-editing techniques to replace this marker with another fluorescent marker in the allelic position. Low rates for homology-directed repair (HDR) in plants have prevented such targeted knock-ins from being efficiently accomplished. Recent developments, however, have provided more efficient approaches with HDR rates being reported at levels as high as 6.3% (Sun et al., 2023), 3.2% (Wang et al., 2023), 9.1% (Miki et al., 2018); and targeted T-DNA integration via *Agrobacterium*-mediated transformation in rice ranging from 4 to 5.3% (Lee et al., 2019). Moreover, constructs encoding a Cas9-VirD2 fusion have succeeded in improving HDR-mediated integration in rice transformation as well (Ali et al., 2020).

An informative marker tool is possible by using the resulting F1 progeny from a cross between parents carrying different fluorescent markers in allelic positions. Haploid cells would express only one fluorescent marker while diploid cells would express both (**Figure 4**). Repressor/activator systems such as the Q-system from *Neurospora crassa* may provide another option to track chromosomal segregation, as the presence of a repressor in a diploid containing an activator would prevent the expression of a marker, but in a haploid, the marker would be expressed due to the absence of the repressor. The opposite effect could also be obtained using only a transcriptional activator in the Q-system. Transcriptional activation also controls the Gal4/UAS system where the presence of Gal4 would lead to marker expression in a diploid while a haploid would be repressed. Both systems have been established as molecular tools in plant systems (Waki et al., 2013; Persad et al., 2020). These systems would still require allelic positioning to be relevant in IVNs, in addition to overcoming false identification with leaky signaling. These site-specific allelic placements of reporter genes could also be achieved using recombinases such as the Cre-lox and FLP-FRT gene-stacking system (Nandy et al., 2015).

Alternatively, a non-allelic bi-fluorescent reporter system may provide a readily available alternative to this process. The non-allelic, hybrid line would only require establishing, two single-locus fluorescent marker lines that would be crossed, and the resulting F_1_, which would carry both markers, can be used for testing meiosis-inducing factors. However, this system has a decreased efficiency caused by a 50% reduction of “informative” artificial gametes, expressing only a single fluorophore compared to the allelic system. However, the speed of development of marker lines provides a relevant strategy for Phase I (**Figure 4**).

### 3.4 RNA sequencing and fusion technologies

Technological advances in RNA sequencing may also contribute to determining meiosis-induction factors in plants. Single-cell RNA sequencing (scRNA-seq) has proven to be an efficient and cost-effective approach to analyzing multiple tissue types in response to treatment (Shin et al., 2019; Shulse et al., 2019). This technology could be used as an assay to assess candidate meiotic induction factors and provide expression data. scRNA-seq with barcoding permits the sequencing of multiple samples through multiplexing, which allows for simultaneous evaluation of multiple treatments and factors (Macosko et al., 2015; Shin et al., 2019; Shulse et al., 2019). Nelms and Walbot (2019) demonstrated the use of this tool to determine gene expression profiles during different stages of meiosis development. Comparative studies of germline and somatic cells have provided insight into differential gene expression in the meiosis of plants (Dukowic-Schulze et al., 2014a; Nelms and Walbot, 2019; Barakate et al., 2021; Dukowic-Schulze et al., 2014b). The availability of reference genes for meiotic processes also provides an opportunity for factors to be tested using quantitative PCR (Ji et al., 2014; Nelms and Walbot, 2019; Garrido et al., 2020). Therefore, these molecular tools could be used to determine the onset of artificial meiosis induction. It has also been considered, that meiosis induction could be assessed by fluorescent fusions with meiosis-specific genes such as the *PRO_REC8_:REC8:GFP* line developed by Prusicki et al. (2019). These methods would provide evidence for specific steps in meiosis and could be scaled for high-throughput investigation. Given this, however, fluorescent-based markers may provide many benefits to tracking and assessing meiosis induction, especially with up-scaling and cost reduction using available commercial instruments, as laborious nucleic acid isolation would not be required.

### 3.5 Statistical approaches for the detection of rare events

The complexity of datasets and inherent variance expected among biological samples would require the optimization of robust statistical analysis methods to detect and discriminate artificial gametes at low meiotic induction rates. The method would rely on analyzing a multitude of data points and determining which factor(s), if any, play a role in meiosis induction. The limit of detection must be possible with induction rates as low as 1%, based on *in vitro* meiosis induction rates found in human cells (Medrano et al., 2016). Hence, large totipotent protoplast populations that can be analyzed are preferred.

**Figure 5:**
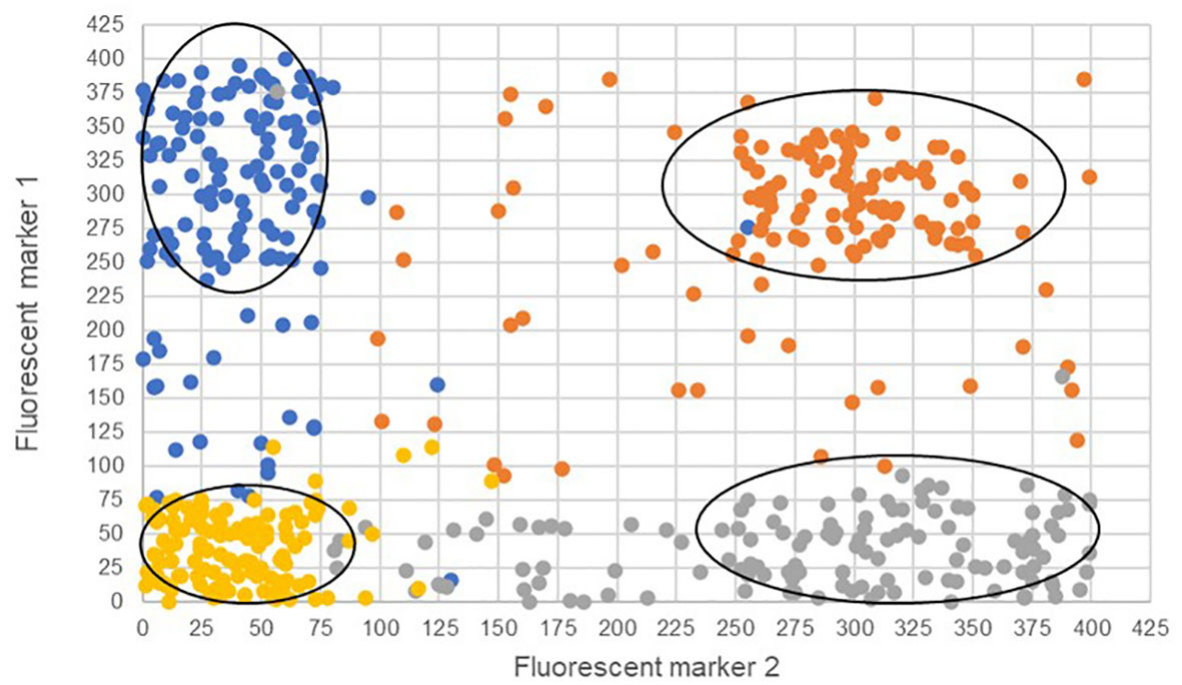
Four theoretical cell populations were produced to simulate mock flow cytometry analysis data with theoretical gating classifications (ovals) for either only fluorescent marker 1 (blue), only fluorescent marker 2 (gray), both fluorescent markers (orange), or the absence of fluorescent markers (gold). The ovals represent potential gating for individual populations. Misclassified cells are depicted as those that have fallen outside the gating ovals or those that have an incorrect fluorescence classification and are a different color than others in the same population.

**Figure 6:**
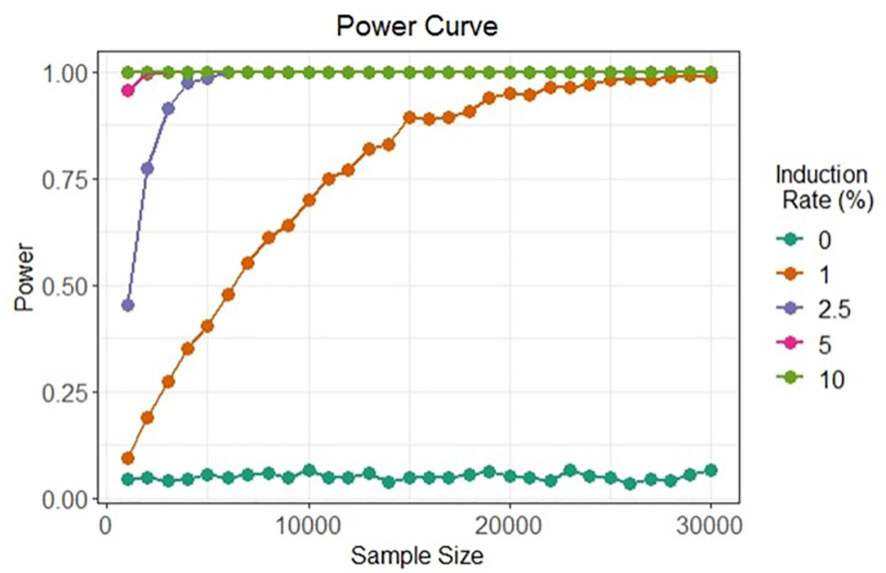
Simulated in silico power for statistical testing of fluorescent cell detection. Each curve denotes the power of the chi-squared test to detect artificial gametes at four different rates of meiosis induction upon the evaluation of different cell sample sizes under possible misclassification by an SVM classifier.

The two bi-fluorescent systems outlined above can use flow cytometry or FACS to detect the different cell populations that show different fluorescence signals. Analysis of fluorescence values can be done using the popular method of “gating” (Adan et al., 2017). Gating is a technique where regions of fluorescence are manually selected to identify events, in our case artificial gametes and diploid cells. **Figure 5** depicts a theoretical gating approach, where a balance between accuracy of cell identification and the number of cells identified must be reached. Cells containing both fluorescent markers (i.e., diploid cells), would show similar fluorescence signals for both markers (i.e., population near the middle of the plot), and cells containing only one of the fluorescent markers (i.e., haploid cells), would predominantly show fluorescence of one of the two markers (i.e., population near either of the two axes). Borges et al. (2012) used a similar FACS method to successfully sort nuclei tagged with either RFP in vegetative nuclei or GFP in sperm nuclei from intact bi-fluorescent pollen, obtaining purity rates as high as 99%. Gating is a potential solution to fluorescent cell discrimination, but the subjectivity in gating may induce unwarranted biases in the follow-up statistical analysis to determine the difference among multiple treatments. Alternative methods for gating could be support vector machines (SVM) or clustering. SVM (Cortes and Vapnik, 1995; Lee et al., 2012) is a supervised machine learning technique that learns from labeled training data and creates hyper-planes that separate the artificial gametes from the diploid cells. Clustering is an unsupervised learning (Lo et al., 2008) for automated gating of flow cytometry data that can estimate the cluster means, covariance matrices, and proportions of each cell type. All these data science tools have non-zero probabilities of misclassification, that is, classifying an artificial gamete as diploid and vice versa, and these misclassification probabilities affect the power of the statistical analyses. Thus, the number of cells needs to be adjusted to account for the loss in power due to misclassifications. **Figure 6** shows a chi-squared test’s power curves under possible misclassifications by SVM for a range of meiosis induction percentages. The number of cells required to detect, for example, a 1% meiosis induction rate with at least 80% power is around 13,000, which is well within the limit of flow cytometry. Further, these results suggest that pooling samples after meiosis induction treatment may provide an efficient approach to testing multiple factors to reduce flow cytometry costs. Pools of interest can then be analyzed more in-depth to identify the factor responsible for artificial gamete induction.

In summary, detection of the few induced gametic cells in a large population of predominantly diploid cells must be supported by a robust statistical framework to provide confidence in factors that result in artificial gametes at low rates, considerations such as this help to define testing procedures and technical limits.

## 4 Discussion

The development of IVNs could greatly benefit plant breeding as a new tool to increase genetic gain. The conservation of meiotic processes in eukaryotes provides evidence of the potential to develop a universal system to induce meiosis for all plant species *in vitro* with only minimal changes to culture conditions. To make progress in IVNs, however, a cost-efficient, high-throughput detection tool must be developed for detecting artificial gametes, which is supported by a robust statistical framework. Such a tool would allow the evaluation of a large number of factors as potential inducers of meiosis. Additionally, as custom molecules for targeted biological processes such as the PROTAC system (reviewed in Békés et al., 2022) become more widely available, opportunities to target genetic factors may be tested more efficiently without needing genetic transformation.

There is much to learn from natural phenomena such as apomixis and parthenogenesis, which may provide insights into approaches that can be reversed in order to induce meiosis. Gene activation technologies paired with increased gene editing capabilities have promise in plant meiosis induction, especially in reversing the effect of a knock-out (Pan et al., 2021; Pan et al., 2022). Additionally, while environmental factors and hormone signaling show clear effects on reproduction, systematic testing of these factors will need to be well-thought out as these factors usually have global consequences on plants. By using liquid based culture systems, factors can be applied easier and in a more uniform fashion, which may further increase the scale of a meiosis induction screening system.

For detection we have proposed a protoplast system and while protoplasts can be isolated easily and in large numbers, which is amenable to the detection systems discussed in this article, protoplast regeneration can be species-dependent and recalcitrant (Hahne et al., 1989; Xu et al., 2022). There may be other technologies that provide different benefits to such a system and should also be explored. Additionally, as new cytometric and microfluidic technologies such as impedance flow cytometry (Heidmann et al., 2016) continue to improve, DNA stains and fluorescent markers may no longer be needed as cells could be detected, quantified, and sorted for downstream manipulation using label-free approaches.

## 5 Concluding remarks and future directions

IVN’s have the potential to change cultivar development in big ways as they can increase genetic gain by decreasing breeding cycle time while also being kept in controlled laboratory conditions. In this review, we have assessed the bottleneck that we believe to be the most limiting at the current state, meiosis induction, but in order to implement and scale IVN’s to efficient sizes, other bottlenecks will need to be overcome. These include the induction and detection of meiosis in crop species, artificial gamete selection, fusion, and subsequent propagation. These bottlenecks will be addressed in subsequent review articles as considerable research is needed. By fully understanding the gaps in knowledge in IVNs, solutions can be more efficiently explored and shared. Progress in single cell analyses, transformation, and sequencing technologies will continue to push IVN’s from ideas to reality.

## Author contributions 

TC drafted and compiled manuscript. DI, SS, PK contributed to writing and editing manuscript. SA finalized figures and editing. SD and EB, contributed to writing and figure development. SH and LD contributed to draft writing. Additionally, BN and TL contributed to draft development and editing. All authors contributed to the article and approved the submitted version.
